# Correction: Urine metabolomic profiles of autism and autistic traits–A twin study

**DOI:** 10.1371/journal.pone.0315559

**Published:** 2024-12-06

**Authors:** Abishek Arora, Francesca Mastropasqua, Sven Bölte, Kristiina Tammimies

The Data Availability statement for this paper is incorrect. The correct Data Availability statement is as follows: The data sets are accessible, after necessary clearances, through the Swedish National Data Service’s (SND) research data catalogue (SND-ID: 2024–444, DOI: 10.48723/6821-pn89).
