# Supplementary material for: Urine metabolomic profiles of autism and autistic traits–A twin study
Source: PLoS One. 2024 Sep 3;19(9):e0308224. doi: 10.1371/journal.pone.0308224 (PMC11371219; doi:10.1371/journal.pone.0308224)
Supplement: Attachment — Submitted filename: RATSS_Reviewer_Comments_Response.pdf [file pone.0308224.s005.pdf]

Reviewer

The metabolomic analysis in twin-cohort to discover urinary biomarker of autism is interesting and of significance. It is valuable to tease out many confounding factors, as gene and environment factors. Although no significant metabolic drivers for autism diagnosis were detected when controlling for other neurodevelopmental conditions. Some nominally changes were found within the twin pairs.

Nevertheless, we have the following questions：

1. For PCA analysis, 9 individuals were outliers and excluded, but no better clustering and explained in further repeated PCA. What is the conceptual basis and meaning for excluding individual?

As rightly stated, following PCA, 9 individuals were designated as outliers and excluded as they were outside the 95% confidence interval ellipses of the scatter plot for the most explanatory principal components (PC1 and PC2, Figure 1A). Further repeated PCAs did not improve clustering and were therefore not included. This rationale and our approach to handling outliers in this study is stated in the manuscript (lines 208 – 213). The removal of the outliers was done based on recommendations in the field (PMID: 38179935). Nevertheless, only 8.57% of the total study participants (N=105) were excluded, which would not have a statistical impact on our findings.

2. For untargeted metabolomics, features identified by the UPLC-MS should be listed and offer more details, as m/z, rt or fragmentation similarity, etc. It is important for exhibiting confidence in annotating metabolites.

The 208 features identified following untargeted metabolomic using UHPLC-MS have been stated in Table S1 and Table S2. The UHPLC-MS analysis and annotation were performed by the Proteomics and Metabolomics Facility of the University of Tuscia, Italy, using their standard established pipeline (lines 114 – 148). This analytical approach has also been utilised in previously published papers (PMIDs 32512190 and 27904735) by members of the core facility. Unfortunately, we were not able to get the *m/z*, rt or fragmentation similarity from the core facility but only the standard annotated metabolites as reported. As the metabolomics analysis

was performed on a clinical cohort, further data cannot be included in this manuscript in lieu of ethical considerations.

3. Urine is an unstable milieu, collection time and method may affect the results. While no information was given in the manuscript. Please explain your consideration of these factors.

Thank you for noting, we have now included more information about how the urine was collected from the participants (lines 110 – 116). We additionally added a sentence about the collection of the urine samples and instability to the manuscript's discussion (lines: 381 – 382).

4. As mentioned in the manuscript, indole-3-acetate is positive associated with autistic traits within the twin pairs. If it is possible to assess the potential of indole-3-acetate as a predictor?

Although, indole-3-acetate is positively associated with autistic traits within the twin-pairs, it would not be appropriate to access its potential role as a predictor due to the confounding effects of participant genetics.

Academic Editor

When submitting your revision, we need you to address these additional requirements.

1. Please ensure that your manuscript meets PLOS ONE's style requirements, including those for file naming. The PLOS ONE style templates can be found at https://journals.plos.org/plosone/s/file?id=wjVg/PLOSOne_formatting_sample_main_body.pdf and https://journals.plos.org/plosone/s/file?id=ba62/PLOSOne_formatting_sample_title_authors_affiliations.pdf

The manuscript has been updated to meet the shared style requirements of PLOS ONE, including the naming of all uploaded files.

2. We note that the grant information you provided in the 'Funding Information' and 'Financial Disclosure' sections do not match.

When you resubmit, please ensure that you provide the correct grant numbers for the awards you received for your study in the 'Funding Information' section.

We have ensured that the grant information provided at the time of resubmission matches that which is included in the manuscript (lines 415 – 422).

3. Thank you for stating the following financial disclosure: "The project was supported by the Swedish Research Council (S.B., and K.T.), Swedish Foundation for Strategic Research (K.T.), the Swedish Brain Foundation – Hjärnfonden (K.T.), the Harald and Greta Jeanssons Foundations (K.T.), Åke Wiberg Foundation (K.T.), Strategic Research Area Neuroscience Stratneuro (K.T.), The Swedish Foundation for International Cooperation in Research and Higher Education STINT (K.T.), and Board of Research at Karolinska Institutet (K.T.). " Please state what role the funders took in the study. If the funders had no role, please state: ""The funders had no role in study design, data collection and analysis, decision to publish, or preparation of the manuscript."
If this statement is not correct you must amend it as needed. Please include this amended Role of Funder statement in your cover letter; we will change the online submission form on your behalf.

The funders had no role in this study and this has been stated in the funding information (lines 420 – 422), as shared in the above-mentioned statement.

4. In the online submission form, you indicated that the mass spectrometry data is accessible from the corresponding author after necessary clearances. The utilised code is available on GitHub (https://github.com/Tammimies-Lab/RATSS-Metabolomics) or available upon request from the corresponding author.
All PLOS journals now require all data underlying the findings described in their manuscript to be freely available to other researchers, either 1. In a public repository, 2. Within the manuscript itself, or 3. Uploaded as supplementary information.

This policy applies to all data except where public deposition would breach compliance with the protocol approved by your research ethics board. If your data cannot be made publicly available for ethical or legal reasons (e.g., public availability would compromise

patient privacy), please explain your reasons on resubmission and your exemption request will be escalated for approval.

The data included in this manuscript pertains to clinical research and cannot be made publicly available for ethical and legal reasons as this would compromise patient privacy.

5. Your ethics statement should only appear in the Methods section of your manuscript. If your ethics statement is written in any section besides the Methods, please delete it from any other section.

The ethics statement is included in the Materials and methods section (lines 86 – 88) and has been removed from any other section of the manuscript.

6. Please include captions for your Supporting Information files at the end of your manuscript, and update any in-text citations to match accordingly. Please see our Supporting Information guidelines for more information: http://journals.plos.org/plosone/s/supporting-information.

Captions for the Supporting information files have been included in the manuscript (lines 437 – 448) and match the in-text citations.
