# Supplementary material for: Urine metabolomic profiles of autism and autistic traits–A twin study
Source: PLoS One. 2024 Sep 3;19(9):e0308224. doi: 10.1371/journal.pone.0308224 (PMC11371219; doi:10.1371/journal.pone.0308224)
Supplement: Attachment — Submitted filename: review PONE.docx [file pone.0308224.s004.docx]

The metabolomic analysis in twin-cohort to discover urinary biomarker of autism is interesting and of significance. It is valuable to tease out many confounding factors, as gene and environment factors. Although no significant metabolic drivers for autism diagnosis were detected when controlling for other neurodevelopmental conditions. Some nominally changes were found within the twin pairs.

Nevertheless, we have the following questions：

1. For PCA analysis, 9 individuals were outliers and excluded, but no better clustering and explained in further repeated PCA. What is the conceptual basis and meaning for excluding individual?

2. For untargeted metabolomics, features identified by the UPLC-MS should be listed and offer more details, as m/z, rt or fragmentation similarity, etc. It is important for exhibiting confidence in annotating metabolites.

3. Urine is an unstable milieu, collection time and method may affect the results. While no information was given in the manuscript. Please explain your consideration of these factors.

4. As mentioned in the manuscript, indole-3-acetate is positive associated with autistic traits within the twin pairs. If it is possible to assess the potential of indole-3-acetate as a predictor?
